# School Satisfaction and Its Associations with Health and Behavioural Outcomes among 15-Years Old Adolescents

**DOI:** 10.3390/ijerph191811514

**Published:** 2022-09-13

**Authors:** Simona Horanicova, Daniela Husarova, Andrea Madarasová Gecková, Andrea F. De Winter, Sijmen A. Reijneveld

**Affiliations:** 1 Department of Health Psychology and Methodology Research, Faculty of Medicine, P.J. Safarik University in Kosice, Tr. SNP 1, 040 01 Kosice, Slovakia; 2 Department of Community & Occupational Health, University Medical Center Groningen, University of Groningen, A. Deusinglaan 1, 9713 AV Groningen, The Netherlands; 3 Institute of Applied Psychology, Faculty of Social and Economic Sciences, Comenius University in Bratislava, Mlynské Luhy 4, 821 05 Bratislava, Slovakia

**Keywords:** school satisfaction, adolescence, hopelessness, health complaints, fighting, truancy

## Abstract

**Background:** Health and behavioural outcomes of adolescents have been shown to be related to school pressure, demands or unfavourable relationships with classmates or teachers. These associations may relate to school satisfaction, but evidence on this is lacking. Therefore, our aim is to explore the associations of school satisfaction with hopelessness, health complaints, fighting and truancy. **Methods:** Data come from the cross-sectional Health Behaviour in School-aged Children study collected in 2018 from Slovak 15-year-old adolescents (N = 816; 50.9% boys). School satisfaction was measured by school engagement and attitudes towards education, grouped as: satisfied (both positive), inconsistent (one positive, one negative) and indifferent (both negative). Hopelessness, health complaints, fighting and truancy were measured using self-report questionnaires. Logistic regression models were used to explore the associations of school satisfaction with hopelessness, health complaints, fighting and truancy separately. **Results:** Indifferent adolescents were more likely to feel hopeless, to frequently experience two or more health complaints, to be involved in a fight and to skip school (odds ratios/95%-confidence interval: 2.57/1.49–4.45; 2.51/1.48–4.25; 1.92/1.02–3.60; and 2.34/1.25–4.40, respectively) than satisfied adolescents. Inconsistent adolescents were more likely to frequently experience two or more health complaints than satisfied adolescents (1.72/1.05–5.79). **Conclusions:** School satisfaction affects adolescents’ health and social behaviour and may threaten their healthy development.

## 1. Introduction

Education is one of the most important tools for gaining the skills necessary for an individual’s contribution to society and for improving young people’s development and health [1,2,3]. Learning and understanding its value from an early age is crucial for further growth, success and prosperity. Schools are the main providers of formal education and their ability to provide education in an efficient way depends on the quality of adolescents’ experiences in them [4], which makes setting a positive attitude towards school among adolescents highly relevant for academic success and achievement [5,6]. Moreover, positive attitudes towards school and education determine the adolescents’ further motivation and involvement in projects [7]. One of the key indicators of adolescents’ attitudes towards school and education is considered to be school satisfaction [8]. The term school satisfaction is just one of the ways used to describe individuals’ relationship with school. Although the use of this term among the literature is not consistent it closely relates to other similar concepts explaining students’ orientation towards school and/or education including school engagement with regards to the attention, curiosity, interest and passion in learning, connectedness and belonging within the school environment [8,9]. School satisfaction itself includes the extent of the experienced mood, happiness, enthusiasm and boredom at school [10,11].

Compared to other European countries, adolescents in Slovakia have been low-ranking in terms of liking school for up to a decade now, but even so, many adolescents still value the education they will pursue [12]. Such a discrepancy between their attitudes or lack of interest and their values regarding school and education may affect their beliefs in school and future success. Moreover, experiencing long-term dissatisfaction due to negative attitudes towards school and/or education may lead to generalized states of unhappiness or hopelessness [13,14], which in turn may lead to much more serious health complaints, including depressive symptoms, high levels of anxiety, dysphoria or apathy [15,16]. Previous research shows that one of the main causes for experiencing hopelessness is the lack of connectedness from people and institutions [17]. Hence, the experienced dissatisfaction with school as an institution may lead to feelings of hopelessness among adolescents which in turn may have severe effects on their mental and physical health.

On top of that, negative school experiences may negatively affect adolescents’ social behaviour and school performance with long-term consequences. Negative experiences at school may lead to a decreased motivation and further result in deteriorated academic progress, achievement and learning outcomes of adolescents [18]. Dissatisfaction at school has also been shown to lead to more violent behaviour, including bullying and involvement in fights [19,20]. Moreover, adolescents who find school non-beneficial, unsupportive or boring tend to avoid it, i.e., be truant [21]. Long-term avoidance-seeking and skipping school may lead to worsened school results [21,22] and may later culminate in an early drop-out, unemployment, or poor opportunities on the labour market [23,24]. These behavioural consequences of dissatisfaction with school might therefore impact adolescents’ academic journey, their opportunities for employability and social and economic mobility [25,26]. Results of previous research regarding school difficulties and their association with health or behaviour are clear and conclusive. Unfavourable outcomes were mostly explored as a result of stress, school pressure, demands for achievement, relationships with peers and teachers or poverty and disorder [14,27,28,29].

Previous research has shown that experiencing dissatisfaction with school increases among boys who come from low affluence families [30]. Moreover, research has shown that boys are also more likely to feel hopeless than girls [31]. Evidence is, however, lacking on how (dis)satisfaction with school because of negative attitudes towards school and education and their incongruity may affect adolescents’ health and behaviour. Therefore, the aim of this study is to explore the associations of school satisfaction with hopelessness, health complaints, involvement in fights and truancy, taking into consideration gender and socioeconomic status. Based on several literature reviews [15,16,17,19,20], we expect that school dissatisfaction will be associated with poor health and behavior outcomes.

## 2. Materials and Methods

We conducted an online cross-sectional questionnaire-based survey on health and health-related behaviour in a nationally representative sample consisting of 11- to 15-year-old school-aged children. For the purpose of this study we used items on attitude towards education that were only asked to 15-years old adolescents. The younger participating children were assumed to not be able to answer this question due to the underdeveloped required ability to think hypothetically and contemplate possible future outcomes [32].

### 2.1. Sample and Procedure

We used data from the Health Behaviour in School-aged Children (HBSC) study conducted in 2018 in Slovakia. This regards a population-representative sample obtained via two-step sampling. In the first step, 140 larger and smaller elementary schools from rural and urban areas from all regions of Slovakia were asked to participate. These were randomly selected from a list of all eligible schools in Slovakia obtained from the Slovak Institute of Information and Prognosis for Education. In the end, 109 schools agreed to participate in our survey (77.9%). In the second step, we obtained data from 8405 adolescents from the fifth to ninth grades of these elementary schools, aged 11 to 15 years old (mean age 13.43; 50.9% boys) [30,33]. In this study, we used data from 15-year-old adolescents (N = 1293), who answered questions regarding their attitude towards education. Moreover, respondents with missing responses were excluded (N = 477), leading to a final sample of 816 adolescents. The excluded respondents with missing answers did not differ from the included ones with regards to SES (Chi square = 1.21, *p* > 0.05) and school satisfaction (Chi square = 2.13, *p* > 0.05). There were differences between excluded and included respondents with regards to gender (Chi square = 18.99, *p* < 0.001) with more boys with missing responses (62.3% vs. 49.8%).

The study was approved by the Ethics Committee of the Medical Faculty at P.J. Safarik University in Kosice (16N/2017). Procedure of approvement included the assessment of the HBSC study protocol which contains the information about the passive consent procedure. Parents were informed about the study via the school administration and could opt out if they disagreed with their child’s participation.

### 2.2. Measures

*School satisfaction* regarded school engagement and attitudes towards education. *School engagement* was measured using the item: “How do you feel about school at present?”, with four-point Likert-type responses (“I like it a lot”; “I like it a bit”; “I don’t like it very much”; “I don’t like it at all”). *Attitudes towards education* were measured using the item “Do you care what kind of education you will have?”, with three-point Likert-type responses (“I care a lot”; “I care about it, but not too much”; “I could not care less”). Both variables were dichotomized into two categories. Next, we created a composite variable of school satisfaction by grouping into three groups: (1) indifferent—adolescents who do not like school a lot and do not care about their education a lot, (2) inconsistent—adolescents who do not like school a lot and care about their education a lot or adolescents who like school a lot and do not care a lot about their education, and (3) satisfied—adolescents who like school a lot and care about their education a lot [30,33].

*Hopelessness* was measured using five items: “All I see ahead of me are bad things, not good things”; “There’s no use in really trying to get something I want because I probably won’t get it”; “I might as well give up because I can’t make things better for myself”; “I don’t have good luck now and there’s no reason to think I will when I get older”; “I never get what I want, so it’s dumb to want anything” with two response options: “I agree” and “I don’t agree” (Cronbach’s α = 0.78). Responses were summed and dichotomized to get two groups of adolescents: those who expressed hopelessness in at least one item and those who did not [34].

*Health complaints* were measured using the HBSC-Symptom Checklist, which assesses the occurrence of eight subjective psychosomatic health complaints, including: headache, stomach ache, backache, feeling low, irritability and bad temper, feeling nervous, sleeping difficulties and feeling dizzy (prevalence is available in the Supplementary Material). The response categories indicated the frequency of the occurence of each complaint within the last 6 months (“rarely or never”, “about every month”, “about every week”, “more than once a week” and “about every day”). We dichotomized the responses for specific health complaints (“rarely or never”; “about every month” and “about every week” vs. “more than once a week” and “about every day”). Moreover, the recurrent multiple health complaints were summed and dichotomized, with two or more complaints at least once a week considered as indicating apparent subjective health complaints [35,36].

*Fighting* was measured using one item: “During the past 12 months, how many times were you in a physical fight?” with five response categories: “I have not been in a physical fight in the past 12 months”; “1 time”; “2 times”; “3 times”; “4 times or more”. The responses were dichotomized into two categories: those who have been involved in the physical fight at least once in the past 12 months and others [35].

*Truancy* was measured by asking: “In the last 12 months did you skip school without a proper excuse for at least one whole day?”. Three response categories (“Never”; “Once or twice”; “Three times or more”) were dichotomized into those who skipped school at least once in the last 12 months and others [12].

*Socioeconomic status (SES)* was measured using the Family Affluence Scale III (FAS-III), which consists of six questions: “Does your family own a car, van or truck?” (No/Yes, one/Yes, two or more), “Do you have your own bedroom for yourself?” (Yes/No), “How many computers does your family own?” (None/One/Two/More than two), “How many bathrooms (room with a bath/shower or both) are in your home?” (None/One/Two/More than two), “Does your family have a dishwasher at home?” (Yes/No), “How many times did you and your family travel out of your country for a holiday/vacation last year?” (Not at all/Once/Twice/More than twice). The sum score was converted into a ridit score ranging from 0 to 1 with the mean (0.5) in the middle of the distribution. Next, we created three tertiles: low (0 to 0.333), medium (0.334 to 0.666) and high (0.667 to 1) socioeconomic position [37].

### 2.3. Statistical Analyses

First, we described the baseline characteristics of the sample using descriptive statistics. Next, we assessed the associations of school satisfaction (independent variable) with hopelessness, health complaints, fighting and truancy (dependent variables), first nod-adjusted and then adjusted to gender and SES using binomial logistic regression models. All analyses were performed using IBM SPSS Statistics 23 for Windows.

## 3. Results

### 3.1. Baseline Characteristics

Table 1 shows the descriptive characteristics of the study sample. Almost 60% of the adolescents reported that they were inconsistent, and nearly 30% of the adolescents reported that they were indifferent. Nearly 35% of the adolescents reported feeling hopeless, and almost 43% of the adolescents reported experiencing two or more health complaints at least once a week. A quarter of the adolescents reported being involved in a fight during the last 12 months, and approximately one-fifth of the adolescents reported skipping school at least once during the last 12 months.

### 3.2. Associations of School Satisfaction with Hopelessness, Health Complaints, Fighting and Truancy

The results of the regression analyses showed that adolescents who reported feeling indifferent towards school and education were more likely to feel hopeless, to experience two or more health complaints at least once a week, to be involved in a fight and to skip school compared to the adolescents who feel satisfied (Table 2). Moreover, adolescents who reported feeling inconsistent with regards to school and education were more likely to experience two or more health complaints at least once a week compared to adolescents who feel satisfied. The results were very similar with and without adjustment for gender and SES and are therefore only shown for the latter.

## 4. Discussion

The aim of this study was to explore the associations of school satisfaction with hopelessness, health complaints, fighting and truancy. We found that indifferent adolescents were more likely to feel hopeless, to experience two or more health complaints at least once a week, to be involved in a fight and to skip school in comparison with the satisfied ones. Moreover, inconsistent adolescents were more likely to experience two or more health complaints at least once a week than the satisfied ones. Our results are consistent with our hypothesis.

We also found that indifferent adolescents, i.e., those who do not like school and do not value education were more likely to feel hopeless and to frequently experience two or more health complaints compared to adolescents who feel satisfied. These findings are compliant with our hypothesis and previous research suggesting that negative experiences at school are associated with generalized states of unhappiness, dysphoric moods and frequent occurrence of compromised health, including anxiety, depression, nervousness or headache and stomach ache [15,16,38]. An explanation may therefore lie in the long-term experienced dissatisfaction resulting from the negative attitudes towards school and education, which might affect their general mindset and beliefs [39], leaving adolescents feeling hopeless, nervous or anxious and eventually result in severely jeopardized mental and physical health. Hence, negative experiences at school may negatively affect adolescents’ health.

Moreover, we found that indifferent adolescents were more likely to be involved in fights and skip school as opposed to satisfied adolscents. This is in line with our hypothesis and it confirms previous research indicating that negative experiences at school are linked to truancy and violent behaviour [19,21]. An explanation may be that adolescents who do not find school interesting and profitable with regards to their needs might be more likely to avoid it and be involved in fights more frequently [40]. Dissatisfaction with school as a result of the negative attitudes towards school and education may thus pose a significant threat to adolescents’ health and behaviour.

We found that the adolescents who felt inconsistent, i. e. those who do not like school but value their education, were more likely to frequently experience two or more health complaints than satisfied adolescents. These findings partially confirm our hypothesis and previous research concerning the associations of negative school experience and health outcomes. An explanation may be that these adolescents experience continuous tension because of the incongruence between their attitudes towards school and education, and that this tension may lead to psychosomatic problems, such as nervousness, anxiety, headaches or sleep difficulties [16,41]. However, our findings suggest that the ambiguity in attitudes towards school and towards education does not affect the behavioural outcomes, including involvement in fights and skipping school. An explanation for this partial agreement with previous research may be that support from parents and teachers contributes to the adolescents’ mindset of growth and their beliefs in own abilities [18,42]. Belief in their abilities subsequently helps adolescents recognize their own potential [18,43], which may add to their valuing of edcuation. Support from parents and teachers may prevent adolescents from negative behavioural outcomes resulting from the incongruent attitudes towards school and education.

### 4.1. Strengths and Limitations

The strengths of this study include the use of a relatively large and representative sample of 15-year-old adolescents and following the Health Behaviour of School-aged children (HBSC) survey protocol, which enables comparison across countries. Next, we used validated and reliable measures allowing for an international comparison.

Some limitations need to be mentioned as well. A first limitation is the relatively large number of excluded respondents due to the missing responses (N = 477). We found differences between excluded and included respondents with regards to gender with more boys with missing responses. We do not, however, expect these gender differences resulting in significant bias, e.g., overestimating of our findings. Second, the study design is cross-sectional which does not allow for the exploration of causality nor reversed causality; using a longitudinal study design might be useful for confirmation. Third, we only explore school satisfaction as a determinant of adolescents’ health issues and other factors affecting health issues need to be considered as well. Finally, we used self-reported questionnaires, which means that responses may have been influenced by social desirability, but we tried to moderate this by using validated measures and administering them in an anonymous environment.

### 4.2. Implications and Recommendations

The results from this study contribute to the knowledge of school satisfaction and its consequences on health and behaviour in adolescence, showing that indifferent adolescents seem to be particularly vulnerable and may require more attention and a thorough screening of their health. For practice, it could be worth focusing on the attitudes towards school and education of 15-year-old adolescents, in particular in case of mental health difficulties. Specific attention for mental and psychosomatic symptoms among adolescents should also be considered for those who seem to be more prone to feel indifferent, i.e., boys experiencing learning difficulties or disruption in the social context and living in a low affluence family, as was shown in a previous study [30]. Practical interventions might also focus on promoting teacher support at school, as it has been shown to help adolescents improve their attitudes towards school and education [33]. Furthermore, international routine monitoring and inquiries may be important in revealing and pointing out countries with any sort of shortcomings such as poor and declining school satisfaction in Slovakia which may draw attention of policy makers and apprise them of possible consequences of this issue.

Future research may employ other indicators of school satisfaction such as satisfaction with other classmates, school grades, school experience, and relationships with teachers, and might include other factors that contribute to (dis)satisfaction with school. Moreover, it should focus on ways to reduce the negative outcomes of school dissatisfaction on adolescents’ health and behaviour. Additionally, considering that the situation of the adolescents who are inconsistent in their attitudes towards school and education is a unique one, further research in this area may add new knowledge. A qualitative approach may help to better understand coping mechanisms and resources as a basis for targeting preventive interventions. This could, e.g., include individual or group interviews with adolescents about their perception and attitudes towards school and education as well as adolescents’ own experiences at school.

## 5. Conclusions

The experienced dissatisfaction at school resulting from the negative or incongruent attitudes towards school and education significantly affects adolescents’ health and behaviour and may later compromise their healthy development and academic and professional journey.

## Figures and Tables

**Table 1:** Descriptive statistics of the sample (Slovakia 2018, 15-year-olds, N = 816).

	n (in %)
**Gender**	
Boys	406 (49.8)
Girls	410 (50.2)
**Socioeconomic status**	
Low	274 (33.6)
Middle	242 (29.7)
High	300 (36.8)
**School satisfaction**	
Indifferent	235 (28.8)
Inconsistent	489 (59.9)
Satisfied	92 (11.3)
**Hopelessness**	
Hopeless	285 (34.9)
**Health complaints**	
two or more health complaints at least once a week	353 (43.3)
**Fighting**	
involved in a fight at least once during the last 12 months	208 (25.5)
**Truancy**	
skipped school at least once during the last 12 months	172 (21.1)

**Table 2:** Association of school satisfaction with hopelessness, recurrent health complaints, fighting and truancy, separately: results from binomial logistic regression (Odds ratios, OR; 95% Confidence interval, CI) (Slovakia 2018, 15-year-olds, N = 816).

	Hopelessness		Health Complaints		Fighting		Truancy	
	Model 1 OR (95% CI)	Model 2 OR (95% CI)	Model 1 OR (95% CI)	Model 2 OR (95% CI)	Model 1 OR (95% CI)	Model 2 OR (95% CI)	Model 1 OR (95% CI)	Model 2 OR (95% CI)
**School satisfaction**								
Indifferent	2.66 (1.54–4.58) ***	2.57 (1.49–4.45) **	2.31 (1.38–3.85) **	2.51 (1.48–4.25) **	2.14 (1.17–3.92) *	1.92 (1.02–3.60) *	2.18 (1.17–4.05) *	2.34 (1.25–4.39) **
Inconsistent	1.49 (0.90–2.50)	1.49 (0.87–2.49)	1.68 (1.04–2.71) *	1.72 (1.05–2.79) *	1.53 (0.86–2.72)	1.57 (0.87–2.86)	1.11 (0.61–2.02)	1.10 (0.60–2.01)
Satisfied (reference)	1	1	1	1	1	1	1	1
**Gender**								
Boys		0.92 (0.69–1.24)		0.49 (0.37–0.66) ***		3.94 (2.77–5.61) ***		0.63 (0.44–0.89) **
Girls (reference)		1		1		1		1
**SES**								
Low		1.46 (1.03–2.08) *		1.48 (1.05–2.09) *		0.87 (0.59–1.30)		0.89 (0.59–1.33)
Middle		1.08 (0.75–1.56)		1.37 (0.96–1.95)		1.01 (0.67–1.52)		0.63 (0.41–0.98) *
High (reference)		1		1		1		1

*** *p* < 0.001; ** *p* < 0.01; * *p* < 0.05; 1 = reference group; SES—Socioeconomic status; Model 1—Association of school satisfaction with hopelessness, health complaints, fighting and truancy, separately, crude effect of school satisfaction; Model 2—Association of school satisfaction with hopelessness, recurrent health complaints, fighting and truancy, separately adjusted for gender and SES.

## Data Availability

The data used in this study are available on a reasonable request from the corresponding author.

## Supplementary Materials

The following supporting information can be downloaded at: https://www.mdpi.com/article/10.3390/ijerph191811514/s1, Table S1: Prevalence of each health symptom occurring at least once a week (Slovakia 2018 15-year-olds, N = 816). Table S2: Prevalence of each health symptom occurring at least once a week stratified by gender and school satisfaction (Slovakia 2018 15-year-olds, N = 816).
