# Supplementary material for: School Satisfaction and Its Associations with Health and Behavioural Outcomes among 15-Years Old Adolescents
Source: Int J Environ Res Public Health. 2022 Sep 13;19(18):11514. doi: 10.3390/ijerph191811514 (PMC9516967; doi:10.3390/ijerph191811514)
Supplement: Supplementary file 1 [file ijerph-19-11514-s001.zip › ijerph-1794267-supplementary.pdf]

Table S1 Prevalence of each health symptom occurring at least once a week (Slovakia 2018 15-year-olds, N= 816)

	n (%)
**Irritability**	303 (37.1)
**Nervousness**	288 (35.3)
**Sleep difficulty**	192 (23.5)
**Backache**	147 (18.0)
**Headache**	131 (16.1)
**Feeling low**	108 13.2)
**Dizziness**	94 (11.5)
**Stomach ache**	71 (8.7)

Table S2 Prevalence of each health symptom occurring at least once a week stratified by gender and school satisfaction (Slovakia 2018 15-year-olds, N= 816)

	**Headache**	**Stomach ache**	**Backache**	**Feeling low**	**Irritability**	**Nervousness**	**Sleep difficulty**	**Dizziness**
				n (%)				
**Indifferent**								
Boys	15 (10.8)	9 (6.5)	25 (18.8)	13 (9.4)	53 (38.1)	48 (34.5)	25 (18.0)	12 (8.6)
Girls	29 (30.2)	16 (16.7)	19 (19.8)	24 (25.0)	445 (46.9)	46 (47.9)	35 (36.5)	25 (26.0)
**Inconsistent**								
Boys	19 (8.4)	10 (4.4)	32 (14.2)	13 (5.8)	77 (34.2)	64 (28.4)	39 (17.3)	14 (6.2)
Girls	58 (22.0)	29 (11.0)	57 (21.6)	50 (18.9)	105 (39.8)	107 (40.5)	75 (28.4)	40 (15.2)
**Satisfied**								
Boys	3 (7.1)	2 (4.8)	7 (16.7)	3 (7.1)	10 (23.8)	9 (21.4)	6 (14.3)	0 (0.0)
Girls	7 (14.0)	5 (10.0)	7 (14.0)	5 (10.0)	13 (26.0)	14 (28.0)	12 (24.0)	3 (6.0)
